# Association between patterns of eating habits and mental health problems in Chinese adolescents: A latent class analysis

**DOI:** 10.3389/fnut.2022.906883

**Published:** 2022-08-05

**Authors:** Xiaotong Li, Mengzi Sun, Nan Yao, Jiaqi Liu, Ling Wang, Wenyu Hu, Yixue Yang, Ruirui Guo, Bo Li, Yajuan Liu

**Affiliations:** ^1^Department of Epidemiology and Biostatistics, School of Public Health, Jilin University, Changchun, China; ^2^Department of Orthopedics, The Second Clinical Hospital, Jilin University, Changchun, China; ^3^Department of Nutrition and Food Hygiene, School of Public Health, Jilin University, Changchun, China

**Keywords:** eating habit, mental health, adolescent, latent class analysis, energy intake

## Abstract

**Objective:**

We aimed to investigate the association between different eating habit patterns and mental health problems among Chinese middle and high school students, and further to estimate the interaction effect of different grouping variables on eating habits.

**Methods:**

One thousand three hundred and forty-eight adolescents from Jilin Province in China were involved in this cross-sectional study. Mental health and eating habits were assessed using General Health Questionnaire and questions on Nutrition Knowledge, Attitude and Practice, respectively. Latent class analysis (LCA) was performed to identify eating habit patterns. Binary logistic regression and generalized linear models were used to explore the association between eating habit patterns, energy-adjusted nutrient intakes and mental health problems. Interaction analysis was performed to analyze the association between eating habits and mental health in different groups.

**Results:**

Based on the LCA results, a 3-class parallel model was identified: 648 adolescents (48.1%) were classified in class-1 “Healthy Eating Behavior/Eating at Home,” 452 adolescents (33.5%) in class-2 “Healthy Eating Behavior/Eating at School” and 248 adolescents (18.4%) in class-3 “Unhealthy Eating Behavior/Random Place.” Compared with class-1, participants in class-2 and class-3 were at higher risk of mental health problems, especially for class-3 (*p* < 0.05). The energy and nutrient intakes by different latent classes showed that adolescents who ate unhealthy had lower daily intake of energy, protein, carbohydrate, fiber, Vitamins and minerals (*p* < 0.05). The interaction between age, sleep duration and different eating habits was statistically significant (*p* for interaction < 0.1).

**Conclusion:**

“Unhealthy eating behavior/random place pattern” was positively correlated with mental health problems of adolescents. The adolescents with health diet were accompanied by fewer mental health problems, especially for that eating at home. And there were interactions between eating habits and age, sleep duration on the mental health problems.

## Introduction

Mental health problems affect 10–20% of young people around the world (1). Adolescence is a period of great and rapid changes in students' physiology and habit (2). Many health-related bad behaviors would appear in this period, which increased the risk of mental health problems (3). Mental health status was closely related to health-related problems of young people, especially the decline of academic performance, poor interpersonal relationship, violence and drug abuse. Mental disorders also placed a heavy burden on young people's mortality, mainly due to significantly higher suicide rates among youth with mental health problems. And suicide was the leading cause of death among people aged 15–24 (1). The factors that affected mental health problems were complex and diverse, such as physical health, unhealthy lifestyle and family factors (3–5).

Although the determinants of mental health were complex, more and more studies showed that unhealthy eating habits were related to mental health (6–8). Teenagers were often exposed to unhealthy foods, such as takeout and high calorie snacks, and their meals were irregular. Research showed that people who regularly ate snacks at midnight (4) and skipped breakfast (9) were more likely to have psychological problems such as depression and increased psychological stress. And some studies pointed out that there were significant differences in dietary nutrition intake between students who ate at home and those who ate at school (10). However, the relationship between dietary patterns including eating locations and mental health status was unclear among adolescents (11–14).

Previous studies on mental health and nutrition of adolescents mainly focused on specific nutrients and foods or specific eating habits. The high correlation between various foods or eating habits made it difficult to attribute the impact to a single independent component, which limited the interpretation and application of the results (15). Due to the presence of dietary patterns, the association between diet and mental health outcomes was simplified and robust. The influence of overall diet on mental health may be more likely to translate into dietary advice and public health information (16). Moreover, most diet studies focus on the food types of “dietary patterns.” For example, a study in Norway divided the eating patterns into three types: varied Norwegian/junk or convenient/snacking (17). However, few studies integrated adolescent eating habit and eating environment into different patterns.

Although various methods were used to cluster different eating patterns, there were still great differences in accuracy. Recently, latent class analysis (LCA) has been applied to nutritional epidemiology (18). Compared with factor analysis, LCA is a human-centered analysis method, which classifies samples into several mutually exclusive categories to explore the latent variables behind the categorical explicit variables with statistical correlation (19). And a Chinese study showed that LCA was more suitable for the study of dietary structure and disease risk than factor analysis (20).

Therefore, this study used LCA to analyze the eating habit patterns of adolescents in Jilin Province of China, to explore the association between different potential categories of eating habits and mental health problems, and further to estimate the interaction effect of different grouping variables on eating habits, so as to provide a scientific basis for the development of targeted health intervention measures.

## Methods

### Data source and study population

This cross-sectional study was questionnaire-based, which was conducted from August 2019 to December 2020 in Jilin Province, China, funded by the Department of Science and Technology of Jilin Province, China. A total of 1,875 adolescents were investigated in this survey. We excluded participants who had missing data on GHQ score or covariates, those who reported extreme total energy intake (outside the mean ± 3 standard deviations [SD]). Finally, a total of 1,348 adolescents were involved in our study, with an average age of 14.49 ± 1.99 years. This study covered students from 6 high schools and 6 middle schools in 9 cities in Jilin Province, and adopted the principle of multi-stage stratified random sampling and the form of face-to-face interview.

The study protocol was approved by the Ethics Committee of Jilin University (batch number: 20180515). All participants provided written informed consent. Research was conducted in accordance with the Helsinki Declaration as revised 1989.

### Data collection

Data collection occurred in two steps. The first step was to measure anthropometric parameters. Body composition analyzer of Tsinghua Tongfang BAC-2A was used to measure body weight and body mass index (BMI). Height measurements (without shoes) were taken with a height ruler. In our study, IOTF criteria for Asian children and adolescents were selected to determine BMI cut-off values of 23 and 27 kg/m^2^ for overweight and obesity (21).

The second step was the questionnaire. Students were asked to complete a nutrition status questionnaire, which was divided into four main parts referred to as follows: Section A was the basic information questionnaire, including gender, age, grade, nation and other information. Section B was the General Health Questionnaire (GHQ), which was originally designed by Goldberg in 1972 to examine the mental health status of respondents in community settings and non-psychiatric clinical settings (22). In this study, the Chinese version of GHQ-20 revised by Li was used to reflect the mental health status of subjects in the past month, including 20 items from three dimensions of self-identity, depression and anxiety, which was a useful scale to measure the mental health of students (23). Each item has a score of 0 or 1, and the sum of self-affirmation, depression, and anxiety sub-score is the GHQ total score, ranging from 0 to 20. The higher the total GHQ score, the worse the individual's mental health. The cut-off point for “case” recognition in GHQ-20 is between 3 and 4, with sensitivity and specificity of 78 and 85%, respectively, compared to the DSM-IL criteria (24). Section C included questions on nutrition knowledge, attitude and practices (KAP). The data we used on eating habits was from the nutritional practices section of the KAP questionnaire, which contained 5 questions, as shown in Table 1. The contents of the questionnaire were formulated on the basis of previous studies (25). Section D was the food frequency questionnaire (FFQ), which was a validated semi-quantitative food questionnaire and included more than 170 kinds of food in 11 categories, including cereals, beans, fungi and algae, vegetables and fruits. Both the reproducibility (*r* value = 0.5–0.8) and validity (*r* value = 0.3–0.6) of the instrument were acceptable, as reported elsewhere (26, 27). The intake frequency of each food should be filled in according to times/day, times/week, times/month, or times/year. Meanwhile, the consumption of each food should be recalled and recorded to obtain the daily intake of each food, and the food intake level and nutrients of the subjects were estimated based on the results of dietary frequency questionnaire. Under the guidance of nutrition experts from the School of Public Health, Jilin University, China, before the widespread use of this questionnaire, quality investigators conducted pre-experiments to verify the feasibility and comprehensiveness of the questionnaire and survey process.

**Table 1:** Participants' responses to questions related to nutritional practices.

**Questions**	**Male (*n* = 643)**	**Female (*n* =7 03)**	**Total (*n* = 1,346)**	***p*-value**
1. Do you have breakfast				0.033
Never	15 (2.3)	24 (3.4)	39 (2.9)	
Sometimes	106 (16.5)	126 (17.9)	232 (17.2)	
Often	122 (19.0)	168 (23.9)	290 (21.5)	
Everyday	400 (62.2)	385 (54.8)	785 (58.3)	
2. What is your main way of eating lunch?				0.045
Take-out food	11 (1.7)	12 (1.7)	23 (1.7)	
Dining hall	324 (50.4)	398 (56.6)	722 (53.6)	
Fast food	39 (6.1)	43 (6.1)	82 (6.1)	
Brown bag	17 (2.6)	28 (4.0)	45 (3.3)	
Eating at home	252 (39.2)	222 (31.6)	474 (35.2)	
3. What is your main way of eating dinner?				0.002
Take-out food	12 (1.9)	12 (1.7)	24 (1.8)	
Dining hall	183 (28.5)	252 (35.8)	435 (32.3)	
Fast food	22 (3.4)	30 (4.3)	52 (3.9)	
Brown bag	6 (0.9)	18 (2.6)	24 (1.8)	
Eating at home	420 (65.3)	391 (55.6)	811 (60.3)	
4. Do you like to eat off-campus street food?				0.036
Like	278 (43.2)	344 (48.9)	622 (46.2)	
Dislike	365 (56.8)	359 (51.1)	724 (53.8)	
5. Do you eat the night snack?				0.001
Everyday	46 (7.2)	21 (3.0)	67 (5.0)	
Often	63 (9.8)	55 (7.8)	118 (8.8)	
Sometimes	350 (54.4)	428 (60.9)	778 (57.8)	
Never	184 (28.6)	199 (28.3)	383 (28.5)	

### Statistical analyses

This study clustered eating behaviors by LCA to form different eating habits patterns of adolescents. LCA was conducted using MPlus 8.3 as indicators for each item of eating habits. LCA is a finite mixed model that identifies meaningful homogeneous groups of individuals in a population given a set of measurement variables. We compared LCA for 2–5 categories, these criteria were used to determine the best model: (1) The lower Bayesian Information Criterion (BIC) or the lower Akaike Information Criterion (AIC) values indicate that the model fits well. (2) The *p*-values corresponding to the Lo-Mendell-Rubin adjusted likelihood ratio test (LMR) and bootstrapped likelihood ratio test (BLRT) indicators reach a significant level (*p* < 0.05), indicating that the models of the k classes are significantly superior to the models of the (k−1) classes. (3) The Entropy value ranges from 0 to 1. When Entropy ≥ 0.80 indicates that the classification accuracy reaches 90%. The higher the Entropy value, the higher the classification accuracy (28).

Continuous variables were presented as mean and standard deviation (SD) or Median (25–75th Percentile). Wilcoxon signed rank test was used for comparison between the two groups. Categorical variables were presented as case and percentage and were compared using Pearson's Chi-square test. We took the total score of GHQ-20 from 3 to 4 as the cut-off point to form a binary variable. Next, we used binary logistic regression to evaluate the association between eating habit patterns and mental health problems. Model 2 was adjusted for age and gender; Model 3 was adjusted for variables in model 2 plus physical activity, sleep duration and passive smoking. Generalized linear models were applied to investigate the comparison of energy and nutrient intakes by latent classes with *post-hoc* pairwise comparisons conducted using the Bonferroni correction. Generalized linear models were controlled for covariates including age, gender physical activity, sleep duration and passive smoking. When interactions were significant (*p* for interaction < 0.1), stratified analyses were performed. *P* < 0.05 was considered statistically significant. The analysis was performed using SPSS24.0.

## Results

The demographic characteristics of the participants were presented in Table 2. In total, 704 (52.2%) adolescents had mental health problems and 644 (47.8%) adolescents did not, of whom 47.8% were male and 52.2% were female. The average age of the sample was 14.49 years old, of whom 59.1% were junior middle school students. And most of the students were Han nationality (83.4%). The proportion of female with mental health problems was higher than that of male adolescents (*p* = 0.006). In addition, compared with the adolescents without mental health problems, most of the adolescents with mental health problems had fewer physical activities, shorter sleep duration and were passive smokers (*p* < 0.05).

**Table 2:** Major characteristics of study population.

**Variables**	**Total (*n* = 1,348)**	**No mental health problems (*n* = 644, 47.8%)**	**With mental health problems (*n* = 704, 52.2%)**	***t/χ^2^***	***p*-value**
Age (Mean ± SD)	14.49 ± 1.99	14.67 ± 2.04	14.71 ± 1.95	−0.347	0.729
Gender (*n*, %)				7.647	0.006
Male	644 (47.8)	333 (51.7)	311 (44.2)		
Female	704 (52.2)	311 (48.3)	393 (55.8)		
Grade (*n*, %)				2.662	0.103
Junior high school	796 (59.1)	395 (61.3)	401 (57.0)		
Senior high school	552 (40.9)	249 (38.7)	303 (43.0)		
Nation (n, %)				0.341	0.559
Han	1,124 (83.4)	533 (82.8)	591 (83.9)		
Minorities	224 (16.6)	111 (17.2)	113 (16.1)		
Physical activity (*n*, %)				19.123	<0.001
More than 4 times a week	388 (28.8)	198 (30.7)	190 (27.0)		
2~3 times a week	723 (53.6)	343 (53.3)	380 (54.0)		
1 time a week	185 (13.7)	93 (14.4)	92 (13.1)		
None	52 (3.9)	10 (1.6)	42 (6.0)		
Sleep duration (*n*, %)				45.628	<0.001
More than 8 h	242 (18.0)	144 (22.4)	98 (13.9)		
6–8 h	776 (57.6)	392 (60.9)	384 (54.5)		
<6 h	330 (24.4)	108 (16.8)	222 (31.5)		
Passive smoking (*n*, %)				8.929	0.003
Yes	332 (24.6)	135 (21.0)	197 (28.0)		
No	1 016 (75.4)	509 (79.0)	507 (72.0)		
Monthly household income (RMB) (*n*, %)				2.247	0.523
<1,000	40 (3.0)	19 (3.0)	21 (3.0)		
1,000~5,000	691 (51.2)	334 (51.9)	357 (50.7)		
5,000~10,000	430 (31.9)	211 (32.8)	219 (31.1)		
≥10,000	187 (13.9)	80 (12.3)	107 (15.2)		
BMI (*n*, %)				4.169	0.124
Normal	974 (72.3)	450 (69.9)	524 (74.4)		
Overweight	255 (18.9)	136 (21.1)	119 (16.9)		
Obese	119 (8.8)	58 (9.0)	61 (8.7)		

*SD, standard deviation; BMI, body mass index*.

The association between eating habits and GHQ scores and their subscales has shown in Table 3. The total score of GHQ, as well as the scores of self-affirmation, depression and anxiety sub-score had statistical significance in different breakfast frequency, preference for roadside stall and frequency of night snack (***p*** < 0.05). Regular breakfast eating was positively associated with mental health. And the mental health status of people who ate take out and fast food for lunch and dinner was poor (***p*** < 0.05). In addition, people who liked to eat roadside stalls and often ate night snack had higher GHQ scores (***p*** < 0.001).

**Table 3:** Nutritional behavior based on GHQ total score, self-affirmation sub-score, anxiety sub-score, and depression sub-score of the sample.

**Variable**	**GHQ total score**		**Self-affirmation sub-score**		**Depression sub-score**		**Anxiety sub-score**	
	**M (P25, P75)**	***p*-value**	**M (P25, P75)**	***p*-value**	**M (P25, P75)**	***p*-value**	**M (P25, P75)**	***p*-value**
Frequency of breakfast									
Never	7.0 (4.0, 12.0)	<0.001	4.0 (2.0, 5.0)	<0.001	1.0 (1.0, 3.0)	<0.001	2.0 (0, 5.0)	<0.001
Sometimes	5.0 (2.0, 9.0)		3.0 (2.0, 5.0)		0 (0, 1.0)		1.0 (0, 3.0)	
Often	4.0 (2.0, 8.0)		2.0 (1.0, 4.0)		0 (0, 1.0)		1.0 (0, 2.0)	
Everyday	3.0 (1.0, 6.0)		2.0 (1.0, 4.0)		0 (0, 1.0)		0 (0, 2.0)	
Lunch way									
Take-out	5.0 (3.0, 10.0)	0.005	3.0 (2.0, 5.0)	0.005	1.0 (0, 2.0)	0.171	1.0 (0, 3.0)	0.289
Dining hall	4.0 (2.0, 7.0)		2.0 (1.0, 4.0)		0 (0, 1.0)		1.0 (0, 2.0)	
Fast food	4.0 (2.0, 8.0)		3.0 (1.0, 4.0)		1.0 (0, 1.0)		1.0 (0, 2.0)	
Brown bag	3.0 (2.0, 8.0)		2.0 (1.0, 4.0)		1.0 (0, 1.0)		1.0 (0, 2.2)	
Eat home	3.0 (1.0, 7.0)		2.0 (0, 4.0)		0 (0, 1.0)		0 (0, 2.0)	
Dinner way									
Take-out	5.0 (3.0, 10.0)	0.037	2.5 (1.3, 5.0)	0.021	1.0 (0, 2.0)	<0.001	0.5 (0, 3.0)	0.638
Dining hall	4.0 (2.0, 7.0)		3.0 (1.0, 4.0)		0 (0, 1.0)		1.0 (0, 2.0)	
Fast food	5.0 (2.0, 9.0)		3.0 (0.3, 5.0)		1.0 (0, 2.0)		1.0 (0, 2.0)	
Brown bag	4.0 (1.3, 8.8)		3.0 (1.0, 4.8)		1.0 (0, 2.8)		1.0 (0, 2.0)	
Eat home	3.0 (1.0, 7.0)		2.0 (1.0, 4.0)		0 (0, 1.0)		0 (0, 2.0)	
Roadside stall									
Like	5.0 (2.0, 8.0)	<0.001	3.0 (1.0, 4.0)	<0.001	1.0 (0, 1.0)	<0.001	1.0 (0, 3.0)	<0.001
Dislike	3.0 (1.0, 6.0)		2.0 (1.0, 4.0)		0 (0, 1.0)		0 (0, 2.0)	
Frequency of night snack									
Everyday	4.0 (2, 10)	<0.001	2.0 (1.0, 5.0)	0.001	1.0 (0, 2.0)	<0.001	0 (0, 2.0)	<0.001
Often	5.0 (2, 10)		3.0 (1.0, 5.0)		1.0 (0, 2.0)		1.0 (0, 4.0)	
Sometimes	4.0 (2.0, 8.0)		2.0 (1.0, 4.0)		0 (0, 1.0)		1.0 (0, 2.0)	
Never	3.0 (1.0, 6.0)		2.0 (0, 4.0)		0 (0, 1.0)		0 (0, 2.0)	

*GHQ, general health questionnaire*.

Model fit indicators for solutions with 2 through 5 classes were summarized in Table 4, and three eating habit pattern classes were chosen. To determine the most optimal classification, we first assumed that there were only two classes for all subjects, and gradually increased the number of classes until we found the best model to fit the data. BLRT significance test showed that increasing the number of classifications might improve the model, but LMR showed no statistical significance for 4-class model, indicating that there was no significant improvement in model fitting between 3-class model and 4-class model. When the model class was 3, LMR and BLRT had ***p*** < 0.05, and the entropy was 0.745. For these reasons, the 3-class model was selected as the optimal models in terms of suitability, simplicity and interpretability for further analysis. This solution created 3 mutually exclusive and collectively exhaustive groups into which an individual was assigned to one class based on their greatest likelihood. The different eating habits conditional probability from the selected eating habit patterns for the three classes was presented in Table 5.

**Table 4:** Fit indices for LCA between 2 and 5 classes.

**Number of classes**	**AIC**	**BIC**	**aBIC**	**Entropy**	**LMR**	**BLRT**
2C	12441.793	12603.191	12504.717	0.758	<0.0001	<0.0001
3C	12288.496	12533.196	12383.897	0.745	0.006	<0.0001
4C	12237.969	12565.971	12365.874	0.796	0.975	<0.0001
5C	12194.428	12606.732	12354.783	0.789	0.843	<0.0001

*AIC, Akaike information criterion; BIC, Bayesian information criterion; aBIC, adjusted BIC; LMR, Lo-Mendell-Rubin adjusted likelihood ratio test; BLRT, bootstrap likelihood ratio test*.

**Table 5:** Latent class conditional probabilities for 3 classes of Nutrition Practices.

	**Class 1 (*n* = 6 48, 48.1%)**	**Class 2 (*n* = 4 52, 33.5%)**	**Class 3 (*n* = 248, 18.4%)**
Frequency of breakfast					
Never	0.013	0.013	0.090
Sometimes	0.123	0.174	0.276
Often	0.153	0.243	0.305
Everyday	0.711	0.570	0.329
Lunch way					
Take-out food	0	0.011	0.064
Dining hall	0.358	0.908	0.302
Fast food	0.063	0.026	0.115
Brown bag	0.052	0.015	0.022
Eat home	0.527	0.040	0.496
Dinner way					
Take-out food	0.002	0	0.081
Dining hall	0.008	0.924	0
Fast food	0.044	0.011	0.073
Brown bag	0.015	0.032	0
Eat home	0.931	0.034	0.846
Roadside stall					
Like	0.234	0.539	0.824
Dislike	0.766	0.461	0.176
Midnight snack frequency					
Everyday	0.019	0.043	0.126
Often	0.016	0.104	0.215
Sometimes	0.513	0.651	0.596
Never	0.452	0.202	0.062

**Figure 1:**
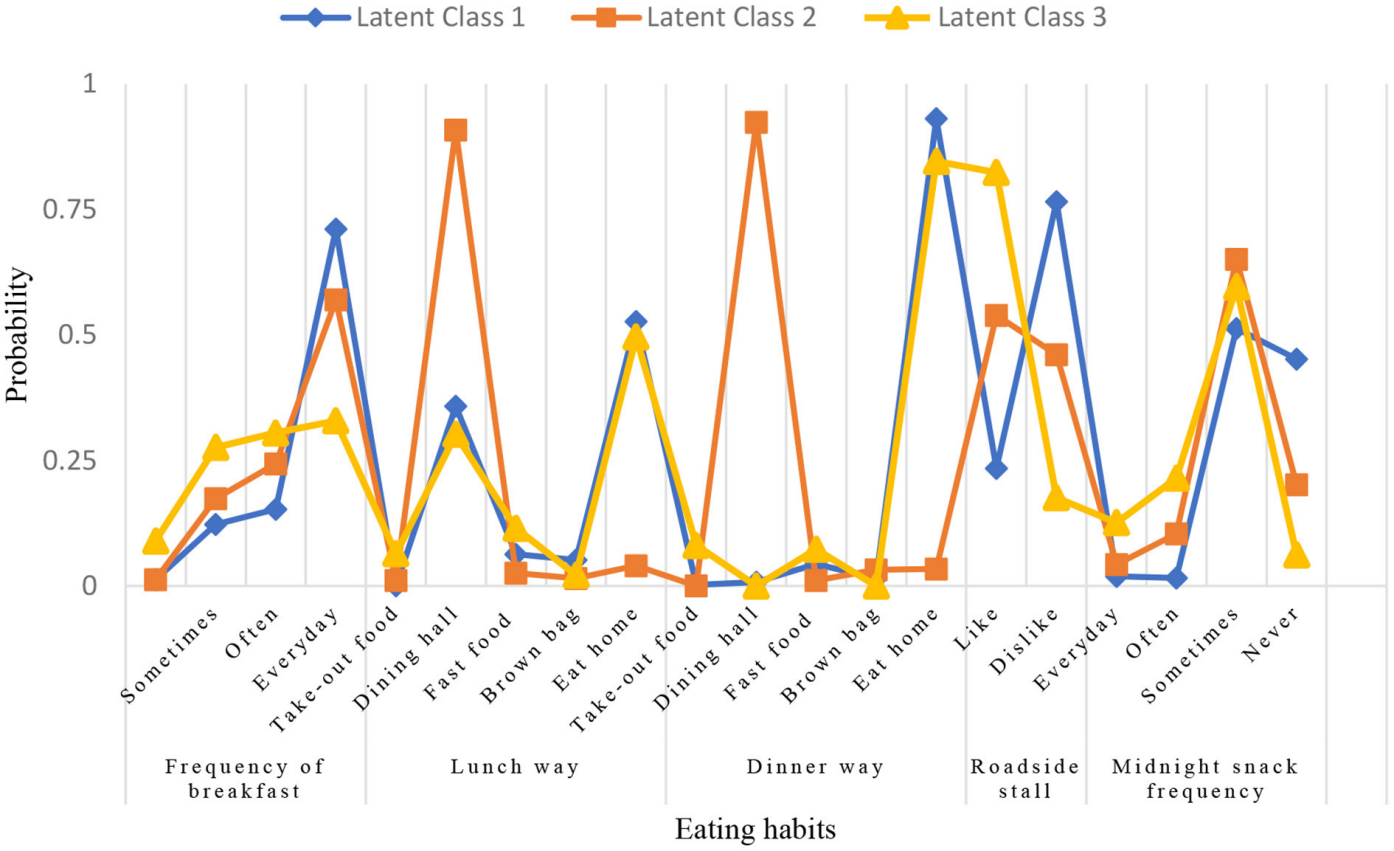
Conditional item probability plots.

Figure 1 showed the project probabilities for each of the three categories of solutions, and we provided subjective labels for each category. Class 1, accounting for 48.1% of the sample (***n*** = 648), was termed “healthy eating behavior/eating at home,” because adolescents in this class had regular breakfast, mostly ate at home for lunch and dinner, and did not often eat roadside stalls and night snacks. The class 2 accounted for 33.5% of the sample (***n*** = 452), and were termed “healthy eating behavior/eating at school.” Adolescents in this class had regular breakfast, and most of them had lunch and dinner in the school canteen. These people did not eat night snacks very often, and nearly half of them didn't like to eat roadside stalls. Class 3, accounting for 18.4% of the sample (***n*** = 248), was termed as “unhealthy eating behavior/random place,” because the teenagers in this class did not have regular eating habits and eating places, most of them skipped breakfast and school meals, preferred roadside stalls, and had a higher rate (34.1%) of eating late-night snacks than the other two classes. This pattern was deemed unhealthy.

Table 6 summarized binary logistic regression analysis using class-1 (healthy eating behavior/eating at home) as the reference. Univariate analysis showed that class-2 (healthy eating behavior/eating at school) and class-3 (unhealthy eating behavior/ random place) were associated with a higher mental health risk (***p*** < 0.05), and the harmful effect of class-3 [adjusted Odds Ratio (OR) = 2.15, 95% Confidence Interval (CI) =1.59, 2.91] on mental health was higher than that of class-2 (adjusted OR = 1.59, 95% CI = 1.25, 2.02). After further adjusting for age, gender, physical activity, sleep duration, and passive smoking in model 3, the ORs (95% CIs) of class-2 (healthy eating behavior/eating at school) and class-3 (unhealthy eating behavior/random place) for mental health problems comparing with class-1 (healthy eating behavior/eating at home) were 1.73 (1.27, 2.38) and 2.23 (1.62, 3.08). The correlation between the two variables did not change (***p*** < 0.05).

**Table 6:** Association between latent class membership and mental health problems.

	**Model 1**		**Model 1**		**Model 1**	
	**OR (95%CI)**	***p*-value**	**OR (95%CI)**	***p*-value**	**OR (95%CI)**	***p*-value**
Class 1: healthy eating behavior/eating at home	Ref		Ref		Ref	
Class 2: healthy eating behavior/eating at school	1.59 (1.25, 2.02)	<0.001	1.78 (1.13, 2.41)	<0.001	1.73 (1.27, 2.38)	0.001
Class 3: unhealthy eating behavior/random place	2.15 (1.59, 2.91)	<0.001	2.38 (1.74, 3.25)	<0.001	2.23 (1.62, 3.08)	<0.001

*OR, odds ratio; CI, confidence interval*.

*Model 1: univariate analysis; Model 2: adjusted for age and gender; Model 3: Model 2 + physical activity, sleep duration, and passive smoking*.

Table 7 presented the comparison of energy and nutrient intakes by different latent classes. After adjustment for multiple covariates, adolescents with an “unhealthy eating behavior/random place pattern” had lower daily intakes of energy, protein, dietary fiber, vitamins and minerals, and higher intakes of total fat (***p*** < 0.05). Compared with “healthy eating behavior/eating at home” adolescents, adolescents who ate at school had higher intakes of minerals (***p*** < 0.05), but there was no statistically significant difference in nutrient intakes (***p*** ≥ 0.05). The results of pairwise comparisons showed that there was no statistically significant difference in the percentage (%) of energy provided by carbohydrates among the three groups (***p*** ≥ 0.05).

**Table 7:** Comparison of energy and nutrient intakes by latent classes.

**Variables**	**Healthy eating behavior/eating at home *(n* = 648)**	**Healthy eating behavior/eating at school (*n* = 452)**	**Unhealthy eating behavior/random place (*n* = 248)**	***p*-value**
Energy, kcal	3543.63 ± 41.39^a^	3509.39 ± 67.30^a^	2931.91 ± 49.30^b^	<0.001
Protein, g	145.70 ± 2.15^a^	146.59 ± 3.49^a^	115.30 ± 2.56^b^	<0.001
Protein (% energy)	16.37 ± 0.14^a^	16.40 ± 0.23^a^	15.66 ± 0.17^b^	0.002
Total fat, g	107.42 ± 1.79^a^	110.56 ± 2.91^a^	96.46 ± 2.13^b^	<0.001
Total fat (% energy)	27.36 ± 0.35^b^	28.96 ± 0.57^a^	29.32 ± 0.42^a^	0.001
Carbohydrate, g	515.20 ± 6.62^a^	509.09 ± 10.76^a^	423.45 ± 7.88^b^	<0.001
Carbohydrate (% energy)	66.23 ± 0.66	59.73 ± 1.08	58.21 ± 0.79	0.140
Fiber, g	29.81 ± 0.63^a^	30.67 ± 1.02^a^	23.81 ± 0.75^b^	<0.001
Vitamin C, mg	212.50 ± 5.93^a^	201.97 ± 9.64^a^	145.50 ± 7.06^b^	<0.001
Calcium, mg	1517.93 ± 38.22^b^	1703.32 ± 62.13^a^	1204.56 ± 45.52^c^	<0.001
Iron, mg	58.08 ± 1.37^b^	63.32 ± 2.23^a^	50.16 ± 1.63^c^	<0.001
Sodium, mg	3615.91 ± 81.70^ab^	3856.61 ± 132.83^a^	3441.56 ± 97.31^b^	0.040
Potassium, mg	4510.14 ± 78.28^a^	4707.14 ± 127.27^a^	3511.53 ± 93.24^b^	<0.001

*Data are the mean ± standard deviation adjusted for age, gender, physical activity, sleep duration, and passive smoking. Different superscript letters indicate pairwise comparisons of the mean using Bonferroni correction, with adjustment for multiple comparisons, between latent classes (p < 0.0167)*.

a, b, c *Represent latent classes with the highest, medium and lowest mean values*.

Table 8 showed the interaction between eating habits and adolescents' mental health problems in different subgroups, and we performed subgroup analyses by different variables. The results showed that adolescents aged <14 years old had a better correlation between eating habits and mental health problems (***p*** for interaction = 0.003). The ORs (95% CIs) of class-3 (unhealthy eating behavior/random place pattern) for mental health problems comparing with class-1 (healthy eating behavior/eating at home pattern) were 2.37 (1.52, 3.69). There was also a significant difference in the association between eating habit patterns and mental health problems among different sleep duration groups (***p*** for interaction = 0.001). In the group with sleep duration of 6–8 h, unhealthy eating behavior was strongly associated with mental health problems (adjusted OR = 2.91, 95% CI = 1.88, 4.51). And we did not find an association between eating habit patterns and mental health in groups with adequate sleep duration (≥8 h) and short sleep duration (<6 h). Furthermore, gender (***p*** for interaction = 0.387) and passive smoking (***p*** for interaction = 0.290) did not significantly alter the association between eating habits and mental health problems.

**Table 8:** Multivariable-adjusted odds ratios for the association between eating habits and mental health problems by subgroups.

	**Class 1: Healthy eating behavior/eating at home *(n* = 648)**	**Class 2: Healthy eating behavior/eating at school (*n* = 452)**	**Class 3: Unhealthy eating behavior/random place (*n* = 248)**	***p* for interaction**
Age				0.003
<14	Ref	1.16 (0.48, 2.83)	2.37 (1.52, 3.69)	
≥14	Ref	1.44 (1.04, 1.97)	1.73 (1.09, 2.73)	
Gender				0.387
Male	Ref	1.68 (1.05, 2.69)	1.93 (1.25, 3.00)	
Female	Ref	1.83 (1.19, 2.81)	2.70 (1.67, 4.38)	
Passive smoking				0.290
Yes	Ref	1.13 (0.64, 2.03)	1.24 (0.59, 2.61)	
No	Ref	2.02 (1.37, 2.96)	2.55 (1.78, 3.64)	
Sleep duration				0.001
More than 8 h	Ref	2.43 (0.75, 7.81)	1.56 (0.77, 3.15)	
6–8 h	Ref	2.35 (1.58, 3.51)	2.91 (1.88, 4.51)	
<6 h	Ref	0.96 (0.51, 1.80)	1.46 (0.72, 2.97)	

*Data are presented as odds ratio (95% confident interval)*.

*Models are adjusted for age, gender, physical activity, sleep duration, and passive smoking except the subgroup variable itself*.

## Discussion

In this study, we used LCA to analyze the association between eating habits and mental health problems among adolescents in Jilin Province. Our study identified three patterns, “healthy eating behavior/eating at home,” “healthy eating behavior/eating at school“ and ”unhealthy eating behavior/random place.” Participants in the “unhealthy eating behavior/random place pattern” had lower daily intake of energy, protein, carbohydrate, fiber, Vitamins and minerals. Unhealthy eating habits were associated with higher mental health risks. The adolescents with health diet were accompanied by fewer mental health problems, especially for that eating at home. Subgroup analysis showed that the association still persisted in age <14 years and participants with less sleep duration (6–8 h).

The incidence of mental health problems among Chinese adolescents in our study was high (52.2%), which was consistent with recent reports (29, 30). There were several factors that may lead to changes in mental health, such as the increase of academic pressure, the change of relationship with classmates or parents, bad behavior (5). Previous reports have pointed out that many people choose to relieve stress by overeating or eating unhealthy food (31). However, studies pointed out that unhealthy diet had no benefit in reducing psychological stress (32). The conclusion was controversial. But study examining eating habit patterns in relation to mental health was lacking. In line with these findings, our study explored the impact of eating habits on adolescents' mental health. We observed that the “unhealthy eating behavior/random place pattern” observed in the present study was associated with higher mental health risk, compared with the “healthy eating behavior/eating at home pattern” and “healthy eating behavior/eating at school pattern,” after adjusting for sociodemographic and lifestyle factors. This finding was consistent with literature reporting a positive association between unhealthy eating behavior and mental health problems.

Epidemiological studies suggested adolescents who didn't eat breakfast were more likely to have elevated stress, depression and emotional distress (7). An earlier study among Chinese teenagers found that college students who ate breakfast almost every day tended to choose healthier food all day (33). A study showed that skipping breakfast was associated with increased cortisol (34), which in turn increased the risk of mental health problems such as anxiety and depression (35). A survey of four provinces in China pointed out that the increased consumption of fast food and sugary drinks had a great impact on teenagers' psychological symptoms (36). The possible explanation was that takeout, fast food and roadside stalls were related to pro-inflammatory diet. Studies pointed out that there was a significant positive correlation between pro-inflammatory diet and the risk of depression (37). In addition, compared with the food at home, fast food and roadside stalls were often less healthy, because these foods contained higher fat, salt and sugar and less nutrients, which were related to various negative health outcomes (38, 39).

Previous studies formed different eating patterns based on dietary intake and eating habits (15–17), but these studies did not consider the impact of eating place on adolescents' mental health. We incorporated eating location into eating patterns, which emphasized that direct food environment might have an impact on adolescents' mental health. Our research results indicated that eating at home had a higher protective effect on adolescents' mental health than eating at school. Children who ate at home had higher intake of core foods (grains, vegetables, fruits, fish, meat, nuts and dairy products) (10). Studies on nutritional intake of children and adolescents in Europe showed that eating at home was associated with higher nutritional intake and lower dietary energy density (40). A study pointed out that regular family meals might promote healthier eating habits, improve parent-child relationship and reduce the risk of depressive symptoms in adolescents (5). Children eating with their parents helped to improve their life satisfaction and emotional stability, and reduced high-risk behaviors such as suicide attempts, antisocial behavior, violence and extreme dieting (14).

Previous research pointed out that limited food and vegetable variety in school cafeterias may lead to inadequate vitamin and mineral intake among teens, and adolescents preferred to buy energy-dense foods in the cafeteria (41, 42). But we did not find similar results in our study. Our results found no significant differences in energy and nutrient intake between home and school meals among adolescents eating healthy eating behaviors. School diets and mental health often involve the interaction of multiple influences and factors. The reason for the poor mental health of adolescents who ate at school may be that most of these students live in dormitories, and they were more likely to encounter problems such as peer influence, school atmosphere pressure, and interpersonal relationships (43, 44). This prompted us to pay more attention to the mental health of students who ate at school.

In the subgroup analysis of this study, we found that the association between unhealthy eating habits and mental health problems was present in younger participants, which was consistent with previous research (11). A potential reason for the age difference may be that younger children were not as good at dealing with mental health issues as older children (45). Furthermore, the relationship was also related to sleep duration. Eating habits had a significant effect on the mental health of participants with a sleep duration of 6–8 h, but there was no correlation when the duration was <6 h. This may be because shorter sleep had a greater impact on mental health status (46), but the relationship between the three need to be further studied. In the findings of this study, girls were more likely to experience mental health problems, and gender differences in mental health risks during adolescence were widely recognized (12). However, we did not find an interaction between gender and eating habits. This was also consistent with results from previous studies showing no signs of gender differences between eating habits and mental health (12, 13).

Our findings have clinical and policy implications. First of all, schools should pay attention to the assessment of students' mental health, and suggest to carry out mental health promotion activities for parents. Educators should promptly identify high-risk groups for early psychological intervention. In addition, we promote strategies for families to eat together. More importantly, schools should strengthen supervision over the sale of unhealthy foods in the surrounding environment, strengthen nutrition and health education for youth schools and families, and cultivate students to form healthy eating habits.

The advantages of this study included the use of LCA as a novel method to obtain three different eating habit patterns according to teenagers' eating places and eating characteristics. The dietary patterns of adolescents were assessed from the overall level, which was more conducive to macro-regulation of diets and more easily translated into dietary recommendations and public health information. And the sample size of the study was relatively large and representative, which can well-reflect the dietary situation of middle school students in Northeast China. Finally, our study explored the association between eating habits and mental health problems stratified by age, sex, and sleep duration, which was conducive to the timely detection of the adverse mental state and take measures.

There were some limitations that should be considered in interpreting the study results. First of all, this study was a cross-sectional study, we cannot judge the causal association between eating habits and mental health status. However, a longitudinal research results did not support the hypothesis of reverse causality (47). Another limitation was that the mental health scale used in this study was not a clinical diagnostic standard scale, so the interpretation of the results should be more cautious. Finally, study outcomes were assessed using self-reported questionnaires and were susceptible to recall bias and misreporting.

## Conclusion

Our results supported the hypothesis that eating habit affected adolescents' mental health. “Unhealthy eating behavior/random place pattern” was positively correlated with mental health problems of adolescents. The protective effect of healthy eating behavior/eating at home on mental health was higher than that of healthy eating behavior/eating at school. And there were interactions between eating habits and age, sleep duration.

## Data availability statement

The original contributions presented in the study are included in the article/supplementary material, further inquiries can be directed to the corresponding author/s.

## Author contributions

YL and XL made the study design. BL, NY, and JL conducted the study. XL, MS, and RG analyzed the data and wrote the manuscript. LW, WH, and YY attended the manuscript revision. All authors agreed with the final manuscript.

## Funding

This work was supported by the Department of Science and Technology of Jilin Province, China (No. 20180623001TC).

## Conflict of interest

The authors declare that the research was conducted in the absence of any commercial or financial relationships that could be construed as a potential conflict of interest.

## Publisher's note

All claims expressed in this article are solely those of the authors and do not necessarily represent those of their affiliated organizations, or those of the publisher, the editors and the reviewers. Any product that may be evaluated in this article, or claim that may be made by its manufacturer, is not guaranteed or endorsed by the publisher.
